# The Quality of Work Index and the Quality of Employment Index: A Multidimensional Approach of Job Quality and Its Links to Well-Being at Work

**DOI:** 10.3390/ijerph17217771

**Published:** 2020-10-23

**Authors:** Georges Steffgen, Philipp E. Sischka, Martha Fernandez de Henestrosa

**Affiliations:** Department of Behavioural and Cognitive Sciences, Institute for Health and Behavior, University of Luxembourg, L-4366 Esch-sur-Alzette, Luxembourg; philipp.sischka@uni.lu (P.E.S.); martha.fernandez@uni.lu (M.F.d.H.)

**Keywords:** quality of work index, quality of employment, well-being, indicators, composite index

## Abstract

(1) Background: Job quality is a multidimensional and elusive concept that is back in vogue among social scientists and policymaker. The current study proposes a new job quality approach that is compared with the European Working Conditions Survey framework and structured with the help of the Job Demands-Resources model. Two new measures of job quality, the Quality of Work Index (QoW) and the Quality of Employment Index (QoE) are developed and validated in three different languages (German, French, Luxembourgish). The QoW is composed of 43 items, focusing on four areas of work—work intensity, job design, social conditions, and physical conditions (subdivided in eleven components)—which are particularly important for employees’ well-being. The QoE is composed of 13 items that cover training opportunities, career advancement, job security, employability, work life conflict, and income satisfaction. (2) Methods: Data were collected via computer-assisted telephone interviews in a representative sample of 1522 employees working in Luxembourg (aged 17–67 years; 57.2% male). (3) Results: Confirmatory factor analysis confirmed the proposed factors structure and scalar measurement invariance for the three different language versions. Internal consistencies were satisfactory for all subscales (Cronbach’s α between 0.70 and 0.87). Correlations and hierarchical regression analyses with different psychological health measures (i.e., burnout, general well-being, psychosomatic complaints, work satisfaction, vigor) and subjective work performance confirmed the construct validity of the new instruments. (4) Conclusions: The QoW and the QoE are globally and on the level of the sub-categories effective tools to measure job quality, which could be used to compare job quality between organizations and different countries. Furthermore, the current study confirms associations between the different components of the QoW and QoE and employees’ health.

## 1. Introduction

In recent decades, the labor market has undergone many changes that were shaped by digitalization, delocalization of production and an increase of non-permanent and part-time work. These changes had also an influence on the working conditions and well-being of employees. The European Union has drawn up different directives (e.g., Europe 2020) that are intended to foster high quality jobs and promote a qualified and healthy labor force that can deal with these new labor market challenges [1,2,3,4]. In order to monitor these labor market changes and their effects on working conditions and well-being of employees and to evaluate the effectiveness of policy interventions it is necessary to develop job quality indicators that also contain psychosocial working conditions indicators. As recent job quality indicators mainly focused on economic aspects [5], there is a need for a new job quality approach. To capture job quality a multidimensional approach is necessary [5,6,7,8]. Governments regulate conditions affecting job quality (health and safety, extension of the work week, paid vacations, etc.) for decades. Improving our knowledge about the dimensions of job quality and integrating what is known in a unified approach seems to be a task well worth to be done [8]. Amongst others it is important to have reliable and valid indicators of job quality.

In the following, we will discuss different job quality approaches with the distinction between quality of work and quality of employment, mainly based on the current research in social sciences on job quality. With the help of empirical data, we will present two new measures that cover quality of work and quality of employment. The different components of the Quality of Work Index (QoW) and the Quality of Employment Index (QoE), with its links to different aspects of well-being and health at work will be investigated. In this way, we follow recent calls to reinvigorate the quality of working life research [9].

### 1.1. Definition and Conceptualization of Job Quality, Quality of Work, and Quality of Employment

There exists no accepted definition of job quality in the scientific literature [8,10,11]. However, many social scientists agree that job quality refers to every aspect of the job that is related to the well-being of the employees. Therefore, job quality can be seen as a multidimensional and elusive concept [8]. Perhaps because of this inherent multidimensionality and elusiveness, there are multiple and relatively diffuse concepts that have been developed in parallel and that tap into the domain of job quality, such as quality of working life, decent work, employment quality, and quality of work [9,10,11,12,13,14,15,16,17]. To make things even more complicated, authors (from different disciplines) referring to the same concept often mean different things (e.g., employment quality [10,13]) and some authors seem to use a few terms interchangeably [12,14,15]. Moreover, different approaches for conceptualizing job quality are documented in the literature [8,11,15]. A first approach proposes to use job satisfaction reported by employees, due to the difficulty of identifying all the aspects affecting job quality and their relative importance. This approach has some important limitations, for instance job satisfaction represents the relationship between the job quality of an employee’s present job and the employee’s idea of what can be reasonably be expected from a job. Thus, employees may get used to poor working conditions and, therefore, have a higher level of job satisfaction compared to employees with better working conditions but also higher expectations regarding their job [10]. Moreover, job satisfaction provides no information on specific working conditions, limiting its use for policy purposes [8]. Thus, job satisfaction is often used as one of other criteria to test the constructed job quality instrument [7]. A second approach uses employees’ surveys to select the components of job quality considered important by themselves. This approach has also some important drawbacks, for instance if employees select different working conditions as being important, comparability can be seriously affected [8,10]. Moreover, employees’ opinion might be conditioned by their current jobs. Therefore, this approach might be useful to identify working conditions that are particularly relevant for a certain working sector, however, it cannot be the sole base to generate a global job quality index. Finally, the third approach considers the theoretical work of social scientists (e.g., economists, sociologists, psychologists) on job quality as a route map to select the relevant dimensions [8]. However, there are different foci between different social science disciplines. While economists emphasize the importance of employment conditions (e.g., wages, career advancement), sociologists and psychologists tend to focus on non-economic work factors such as intrinsically meaningful or challenging work, and in particular on the ‘goodness’ of work when considering job quality [7,8]. These different approaches resulted in a plethora of initiatives aiming at measuring job quality [8,16,17]. Munoz de Bustillo et al. and others [8,13] decompose job quality into two broad areas: quality of work and quality of employment. Quality of work refers to the ways and conditions under which the activity of work can affect the well-being of employees, often focusing on the job content, the work conditions and environment. Quality of employment describes all aspects of a job that are related to the employment relation (e.g., career advancement, work life balance [8]).

### 1.2. Approaches to Measure Job Quality

#### 1.2.1. Existing Job Quality Indicators

Muñoz de Bustillo and colleagues [8,18,19] and others [20] presented overviews of the major international frameworks on job quality indicators. They showed that these different indicators are designed based on different research tradition and from scientists from different disciplines (e.g., Sociology, Political Science, Economics), and are, thus, quite diverse. They vary regarding the number of indicators (from six to over one hundred) [21,22], the focus of the indicators (work quality, employment quality) [23,24,25], the design (cross-sectional, longitudinal) [21,26,27,28]. Further, Muñoz de Bustillo et al. [19], as well as Cazes et al. [20] presented a more extensive summary of the major national and international frameworks, for example from the European Commission [29], UNICE [30], Leschke et al. (ETUI) [23], EMCO [31], ILO [32], Eurofound [4,33], and UNECE [34]. These different frameworks apprehend 4–11 different areas, where the nature of the indicators is mostly objective with only few self-reported, subjective indicators. Often a composite index is not available. Thus, most of these frameworks cover multiple dimensions and rely on numerous indicators of different nature. Additionally, different national initiatives, as for example l’Enquête Conditions de Travail of the French Ministry of Labour, the National Working Condition Survey (NES) in the Netherlands and the Belgian Four-A model [35] show the same problems [20,36]. Additionally, different European surveys providing information on job quality (European Social Survey; International Social Survey Programme; Eurobarometer; Gallup Work Poll; European Quality of Life Survey; EU-LFS AHMs; see [20]) comprise mainly the same components. These components are used by different researchers to define specific indices using the same data set but choosing and analyzing different dimensions or variables of one or more surveys [19,24].

Moreover, many authors have emphasized the outstanding role of the European Working Conditions Survey (EWCS) and its indicators in the context of job quality research [8,19,37]. In the 6th wave of the EWCS Eurofound [33] defined job quality as composition of seven dimensions, representing largely the most important job aspects for well-being of the employees: (1) work intensity (quantitative demands, pace determinants and interdependency, emotional demands), (2) working time quality (duration, atypical working time, working time arrangements, flexibility), (3) physical environment (posture related, ambient, biological, and chemical), (4) social environment (adverse social behavior, social support, management quality), (5) skills and discretion (cognitive dimensions, decision latitude, organizational participation, training), (6) prospects (employment status, career prospects, job security, downsizing) and (7) earnings. From a conceptual perspective, the EWCS is the most convincing and extensive concept. The conceptual framework of the EWCS aims to cover a maximum of areas and indicators of job quality identified in research. The dimensions were selected based on their proven impact on health and well-being of employees [33].

#### 1.2.2. Limitations of Existing Job Quality Indicators

As documented in this overview there exists a multitude of conceptualizations and approaches to measure job quality. However, up to now there is still no general agreement about the dimensions or the measurement of the single dimensions of job quality. Muñoz de Bustillo et al. [8,18,19] noted some limitations of the previous job quality indicators. Some of them mixed job quality and labor market indicators in a global measure that might lead to misinterpretations. Furthermore, they noted that certain working conditions that had been identified as important determinants of employee’s well-being (e.g., work intensity/time pressure) are not assessed in most of their reviewed indicators. Moreover, some indices mix job components with possible outcomes, e.g., job satisfaction. Additionally, as job quality is often not clearly defined, the aim of a job quality index is also often not clearly stated. For instance, does it include working conditions that are mainly related to employees’ performance or working conditions that are mainly related to well-being?

However, besides the conceptual issues of many job quality instruments mentioned in the overviews, additional problems of these instruments may be pointed out that in part also apply to the EWCS [16]. First, many job quality questionnaires have been developed relatively atheoretically, thus, restricting theoretical progress. Second, many of these questionnaires are lacking profound tests of psychometric properties (e.g., test of factor structure or criterion validity). Moreover, Piasna et al. [16] (p. 176) emphasized that “(i) n the literature on job quality, there has been little discussion of the most economical or ‘short-form’ way to measure it on a large scale”. Third, most of these instruments were not tested for measurement invariance across different language versions, a required condition to allow for meaningful comparisons across different language contexts [38].

The discussion of the existing job quality indicators has revealed that despite the current availability of several indices of job quality, there is still a need of a worker-oriented, individually constructed and theoretically grounded job quality indicator with measurement invariant language versions that adapts the conceptual framework of the EWCS in order to measure and monitor the evolution of job quality [8,18]. Thus, the aim of the current study was to develop two new job quality indices that fulfil these criteria. As psychosocial working conditions play an important role regarding well-being, job quality approaches should also consider work psychology theories and studies that emphasize the importance of psychosocial working conditions. For instance, Humphrey et al. [39] employed a meta-analysis to test the influence of different working characteristics on well-being (anxiety, stress, burnout, overload) amongst others. They found that motivational characteristics (e.g., skill variety), social characteristics (e.g., social support) and work context characteristics (e.g., physical demands) explained a substantial amount of variance in different well-being dimensions (*R*^2^ between 0.20 and 0.64).

### 1.3. A New Job Quality Approach

#### 1.3.1. Theoretical Foundation

In work psychology there exists a multitude of theories that try to explain the link between working conditions and different well-being dimensions. However, many of them include only a restricted number of working conditions, such as the job demand control model [40] that only includes work overload and autonomy, although it was later expanded to also include social support [41]. The job demands resources (JD-R) model [42] on the other hand does not limit its focus on specific working conditions [42]. It divides work characteristics into two broad categories, i.e., job demands and job resources [42,43]. Job demands refer to all “physical, psychological, social, or organizational aspects of the job that require sustained physical and/or psychological (cognitive and emotional) effort or skills” [42] (p. 312) and are, thus, related with psychological and/or physiological costs. Job resources, on the other hand, refer to all physical, psychological, social, or organizational aspects of the job that reduce job demands and/or their related psychological/physiological costs, stimulate learning, development and personal growth, are functional to achieve work goals [42,43]. Therefore, resources are not only needed to deal with job demands, but also valued in their own right, as they allow employees to achieve or protect other resources [42]. In addition, they can be located at four different levels: (a) organization at a large level (e.g., career opportunities), (b) interpersonal level (e.g., supervisor and co-worker support), (c) organization of work (e.g., participation in decision making), and (d) organization of task (e.g., performance feedback) [44]. The JD–R model makes predictions about positive and negative health outcomes based on certain job characteristics. Moreover, model proposes that two distinct psychological process underlie the development of job strain and motivation [42,43]. Whereas job demands are presumed to initiate a health-impairment process, thereby leading to employees’ exhaustion and burnout, job resources are expected to have a motivational potential, leading to high work-engagement and increased performance (i.e., motivational process [42,43]). Previous research identified work-home interference, work overload, harassment, emotional demands and physical demands as the most detrimental job demands, whereas relationship with supervisor, social support, feedback and autonomy have been found to be the most beneficial job resources in terms of well-being [42,43,45,46]. Thus, a good system of job quality indicators should assess the most important job demands and job resources that employees encounter at work.

#### 1.3.2. Methodological Choices

A set of methodological choices that have been made prior to the process of the selection of the dimensions to be included in the indices will be presented first [19]. Thus, the modelling of the two job quality indices (i.e., Quality of Work and Quality of Employment) were guided by the following criteria:

- The selection of working conditions is based on the theoretical and empirical work of social scientists.
- The indicators are defined, constructed and anonymously computed at the individual level,
- Only indicators that are relevant for all working sectors were included,
- A composite index is created based on a system of aggregated indicators (on the basis of equal weights),
- The interference with the central, tailor-made concept EWCS [33] is given (see Table 1),
- The collected data is based on self-assessments of employees (self-reported, mostly subjective),
- The main job aspects contribute clearly and directly to employees’ well-being (outcome of job quality),
- The theoretically based differentiation between job demands and job resources is respected,
- Only issues which are related to job quality (eliminating labor market access, the distribution of disposable income, etc.) were considered.

The scientific literature was screened for job characteristics that (a) are in line with the most recent conceptualization of job quality, (b) affect employee’s well-being and/or health, and (c) are in line with the assumptions of the JD-R model. This approach ensures that the instrument covers the most important job characteristics that affect well-being and/or health while at the same time being concise. The development of the instrument was carried out in collaboration with experts from the Luxembourg Chamber of Labor (see Appendix Table A1, Table A2, Table A3 and Table A4 for all items).

#### 1.3.3. Quality of Work

The QoW is compiled of four different areas of quality of work subdivided in two to three dimensions:

(a) Job design (participation, feedback, autonomy);
(b) Work intensity (mental demands, time pressure, emotional demands);
(c) Social conditions (social support, competition, mobbing); and
(d) Physical conditions (risk of accidents, physical burden).

The different dimensions of job design can be regarded as job resources. Participation refers to the involvement of employees in decision-making processes. Previous research has shown that this form of participation is linked with less role stress (e.g., role conflict, role ambiguity) [47], perceived supervisor and organizational support [48], job satisfaction, and skill use and skill enhancement [49]. Feedback reflects the degree to which other organizational members (i.e., colleagues, supervisors) provide information about the work output. It has been meta-analytically linked with burnout and engagement [50]. Autonomy reflects if an employee has ample opportunities to do his/her work autonomous (i.e., decide when and how to do the work as well as the content and order of tasks). It is one of the most often researched job resources [51] and has been meta-analytically linked with burnout [52] and work engagement [53].

The different dimensions of work intensity are job demands that are also related to employee’s well-being. High mental demands can decrease well-being, when no recovery takes place [54]. It has been linked with psychological ill health [55]. Time pressure has been meta-analytically linked to reduced well-being [56], also on a day-to-day level [57] and might also lead to a lack of psychological detachment [58]. Emotional demands has been meta-analytically linked with reduced well-being and job attitudes [59,60].

Additionally, a plethora of studies has shown that social conditions have a strong influence on employee’s health, attitude and behavior. One of the most often studied condition is social support [51]. Social support represents a job resource that reflects the degree to which an employee gets advice and assistance from others when needed. Social support has various effects of well-being as it reduces the experienced strain, mitigates perceived stressors and buffers the stressor-strain relationship [61]. In contrast, competition and mobbing can be seen as special job demands. Competition has been linked to workaholism [62], and, thus, might also have an influence on employee’s well-being. Workplace mobbing refers to a situation, where the employee is being exposed to repeated negative and/or hostile acts from people at work that are experienced as annoying and difficult to defend against [63]. Meta-analytical results showed various detrimental effects for the targeted employees’ well-being and work-related attitudes and behavior [64].

Finally, physical conditions can be seen as job demands that are related to well-being and health. Risk of accidents and physical burden have been linked to well-being and physical health [65,66,67].

#### 1.3.4. Quality of Employment

Different employment conditions (training opportunities, career advancement, job security, employability, work life conflict, income satisfaction) were measured to get an indicator of the quality of employment. Training opportunities have been linked with job satisfaction [68], increased work engagement [69], and reduced turnover intentions [70]. Career advancement has been linked with higher job satisfaction [71], higher affective commitment and higher work engagement [72], as well as reduced turnover [73]. Job insecurity has been meta-analytically linked to various negative outcomes [74]. Furthermore, employability is also linked to well-being [75]. Moreover, low work life balance (i.e., work life conflict) has been found to be related with strain and poor psychological health [76,77]. Finally, income is an important concern for employees. However, what seems to be more important for well-being and job satisfaction than income is satisfaction with income that is affected by the discrepancy of income that employees think they should receive and their actual income [78]. Indeed, research has shown that high income does not improve emotional well-being [79] and that rank of income, not income per se, affects life satisfaction [80]. Therefore, income satisfaction can be seen as key contributor to job satisfaction [78].

#### 1.3.5. Well-Being Dimensions

To validate the newly developed instrument we assessed different well-being and health measures, work satisfaction, vigor and subjective work performance. We used three measures of well-being and health that are interrelated but tap into different aspects of well-being. These are burnout, general well-being, and subjective physiological health problems. Burnout is a work-related well-being construct that is related to several negative consequences (e.g., anxiety, depression, health problems) and is also associated with turnover intentions, organizational commitment, and work satisfaction [52]. Job demands and resources have often been linked to burnout [52]. Furthermore, as work plays a central role in many employee’s life, job quality may also have an influence on the general well-being [81]. Moreover, we included a measure of physiological health problems as job dimensions can also have an influence on physical health symptoms [82]. We also included work satisfaction that is sometimes used as an overall indicator of job quality [8] and that is strongly interrelated with lateness, absenteeism, turnover, organizational commitment, performance, and well-being [83,84,85]. Additionally, we included vigor that has been considered as direct opposite of the burnout subdimension of exhaustion [86]. Thus, we captured the full continuum of employee’s energy and mental resilience [87]. Finally, we assessed (subjective) work performance, to extend the nomological network of the new questionnaire.

## 2. Methods

### 2.1. Measures

The items of the QoW and QoE were mainly newly developed, but we also used some existing items (e.g., [88]) or oriented us on existing questionnaires (e.g., the Work Design Questionnaire [89]; Copenhagen Psychosocial Questionnaire [90]) and adapted the original wording to fit the context of a CATI survey better. During the development of the items, we followed several principals. We sought to create items that reflected the construct definition but were also distinct enough from other constructs. Furthermore, we choose response scales with only five answer categories to reduce cognitive demands of the interviews [91]. Additionally, as our aim was to keep our instrument as short as possible, we only developed between two and five items for each scale. Short scales have the advantage to put less burden on respondents [92] and give researchers the opportunity to assess more constructs [93]. Lately, many researchers have called for short scales to assess specific constructs for general survey research and there are many examples for well-validated ultra-short scales [92].

#### 2.1.1. Quality of Work Index

Unless specified, a five-point Likert response format ranging from 1 (= *to a very low extent*) to 5 (= *to a very large extent*) was used. The area job design contained three dimensions. Participation was measured by two items reflecting if an employee has ample opportunities to be involved in the decision-making process. Feedback was measured by two items reflecting if an employee receives feedback from his/her superior and colleagues. Autonomy was measured by four items reflecting if an employee has ample opportunities to do his/her work autonomously/or in an autonomous manner.

The area work intensity also included three dimensions. Mental demands was measured by two items reflecting if an employee is doing intellectually demanding work. Time pressure was measured by two items asking if work is done under pressure. Emotional demands was measured by two items reflecting if an employee is doing emotionally demanding work. For the items measuring time pressure and emotional demands a five-point Likert scale ranging from 1 (= *never*) to 5 (= (*almost*) *always*) was applied.

The area social conditions also comprised three dimensions. Social support was assessed by three items measuring if an employee gets social support from others at work. Competition was assessed by four items measuring if an employee competes with others at work. Mobbing was assessed with the Luxembourg Workplace Mobbing Scale (LWMS) [94,95] that contains five items (“criticized”, “ignored”, “absurd duties”, “ridiculed”, “conflicts”). Employees were asked to indicate how often they encounter each situation on a five-point Likert scale, ranging from 1 (= *never*) to 5 (= (*almost*) *always*).

The area physical conditions consisted of two dimensions. Physical burden was measured by two items asking if employees are confronted with physical burden. More specifically, participants were asked to indicate the degree to which each item applied to them on a five-point Likert scale, ranging from 1 (= *never*) to 5 (= (*almost*) *always*). Risk of accident was measured by two items asking if employees are confronted with a risk of accident at the workplace.

To calculate the QoW, the scales mental demands, time pressure, emotional demands, competition, mobbing, physical burden and risk of accident were recoded so that higher levels correspond to more favorable working conditions (e.g., less mobbing exposure). The QoW is then created by calculating the mean of each eleven scales. All QoW items can be found in Table A1 in the Appendix A.

#### 2.1.2. Quality of Employment Index

Again, unless specified, a five-point Likert response format ranging from 1 (= *to a very low extent*) to 5 (= *to a very large extent*) was used. Training opportunities was measured by two items asking if employees are involved in formation. Career advancement was measured by two items asking if employees are getting promoted by the employer. Job security was measured by two items asking the extent to which employees consider their job to be safe. Employability was measured by two items. Participants were asked to indicate on a five-point Likert scale how difficult they would consider finding a new job (1 = *not difficult at all*, 5 = *very difficult*). Work life conflict was measured by three items (1 = *never*/*not difficult at all*, 5 = (*almost*) *always*/*very difficult*). Finally, income satisfaction was measured by two items asking if employees are satisfied with their income (1 = *to a very low extent satisfied*, 5 = *to a very large extent satisfied*).

We also calculated a Quality of Employment index (QoE). The scale work life conflict was recoded so that higher levels correspond to less experienced work life conflicts. The QoE is then created by calculating the mean of each six scales. The QoE items can be found in Table A2 in the Appendix A.

#### 2.1.3. Long Work Week and Atypical Working Hours

Working hours per week and atypical working hours were assessed with two open-ended questions. We used the cutoff criteria from the EWCS and coded employees, which worked 48 or more hours per week, as having a long work week (=1) and employees, which worked 47 or less hours per week, as not having a long work week (=0). Furthermore, if an employee stated that (s)he worked more than four days a month in the evening, at night, or at the weekend (s)he is coded as having atypical working hours (=1) and 0 otherwise (see Appendix Table A3).

#### 2.1.4. Well-Being Dimensions

In order to investigate whether the quality of work and quality of employment dimensions can be used to predict employees work satisfaction, well-being and performance, we applied different outcome measures. In line with previous research, we focused on three well-being dimensions: burnout, general well-being and subjective physiological health problems. Performance measures comprised vigor and subjective work performance. The respective items are included in the Appendix Table A4. The three-item work satisfaction scale assessed global judgment of work satisfaction, as well as employee’s satisfaction with important work characteristics, such as work climate and working conditions. The response scale was a five-point Likert scale ranging from 1 (= *to a very low extent satisfied*) to 5 (= *to a very large extent satisfied*). Burnout was assessed with six items of the work-related burnout subscale of the Copenhagen Burnout Inventory [96]. This subscale taps into the domain of work-related emotional exhaustion [96]. The response scale was a five-point Likert scale ranging from 1 (= *never*/*to a very low extent*) to 5 (= (*almost*) *always*/*to a very large extent*). We assessed general well-being with the WHO-5 wellbeing index, a well-validated, brief, general index of subjective psychological well-being [97,98,99]. The response format ranged from 1 (= *at no time*) to 6 (= *all the time*). Subjective physiological health problems were assessed with a seven-item index (i.e., general health problems, headaches, heart problems, back problems, joint problems, stomach pain, sleeping problems). The response scale ranged from 1 (= *never*) to 5 (= (*almost*) *always*). Vigor was assessed with the three-item subscale of the short Utrecht Work Engagement Scale (UWES-9) [86]. Vigor was included as it represents the direct opposite of the core burnout dimension of exhaustion [86] that is assessed with the Copenhagen Burnout Inventory [96]. The response format ranged from 1 (= *never*) to 5 (= (*almost*) *always*). Finally, subjective work performance was assessed with a two-item scale. The response format ranged from 1 (= *never*) to 5 (= (*almost*) *always*).

### 2.2. Translation Process

The questionnaire was developed in three languages (Luxembourgish, French, Germany). First, the items were developed in Luxembourgish by two native speakers, who were also proficient in French and in German (i.e., trilinguals). In a second step, they created the French and the German versions from the original Luxembourgish items. With this approach, we followed several recommendations outlining the importance of taking into account the cultural context during the translation process [100]. Likewise, our aim was to avoid, biases a single translator might have introduced [101]. After the initial translation the questionnaire was tested for comprehension and semantic meaning by five native speakers (in each language). They discussed and refined the translation and generated the final version of the questionnaire.

### 2.3. Data Collection Procedure

In a next step, the questionnaire was tested in a representative sample of employees working in Luxembourg. Data for the present research were entailed via computer-assisted telephone interviews (CATI) in 2017. A dual-frame approach of landline and mobile phone numbers [102] were used to contact employees working in Luxembourg (i.e., Luxembourgish residents and commuters from France, Belgium and Germany). The survey was conducted according to the Declaration of Helsinki (i.e., voluntary participation, participants were free to withdraw their consent at any time throughout the interviews without negative consequences for them). All data reported in the present research are cross-sectional. This project was implemented by the University of Luxembourg in collaboration with the Luxembourg Chamber of Labor [103].

### 2.4. Sample

The sample consisted of 1522 employees working in Luxembourg. Included were Luxembourg residents (60.0%, *n* = 913) and commuters from France (19.8%, *n* = 301), Belgium (10.4%, *n* = 159), and Germany (9.8%; *n* = 149), who received wages for work with at least 10 h of work per week. People doing unpaid voluntary work or internships were excluded from the sample. The sample is representative in terms of employees’ state of residency in Luxembourg. About 43.5% (*n* = 662) answered the Luxembourgish, 42.6% (*n* = 649) the French and 11.3% (*n* = 172) the German version of the questionnaire. The interviewees’ age ranged from 17 to 67 years (*M* = 46.2, *SD* = 9.0). About 37.3% (*n* = 567) of participants had an academic degree. Most participants worked as professionals (28.3%, *n* = 426) followed by technicians and associate professionals (25.0%, *n* = 377), clerical support workers (12.4%, *n* = 186), craft and related trades workers (10.0%, *n* = 151), service and sales workers (9.7%, *n* = 146), managers (5.0%, *n* = 75), plant and machine operators (4.3%, *n* = 64), elementary occupations (4.3%, *n* = 64), skilled agricultural, forestry and fishery workers (0.9%, *n* = 14), and armed forces occupations (0.1%, *n* = 2). Table 2 shows the sample characteristics differentiated for the language versions. While the subsamples have similar characteristics to the total sample, there are some differences. As expected, there are differences regarding the nationalities of the employees who have chosen to answer the different language versions of the questionnaire.

### 2.5. Statistical Analyses

At first, the item characteristics (mean, standard deviation, skewness, kurtosis, percent missing) were analyzed. For further analyses (except for the confirmatory factor analysis), multiple imputation (with five imputed datasets) with predictive mean matching [104] was used to account for missing values. Parcel summaries of scales [105] and all other analysis variables were included in the imputation model with the assumption that missing values are missing at random. Analyses run on each imputed dataset were pooled according to Rubin’s rules [106] and the D1 (multivariate Wald test) statistic was used for multi-parameter inference [104]. Given that the indicators’ multivariate distribution has a strong influence on confirmatory factor analyses’ (CFAs) estimation results, we calculated Mardia’s multivariate skewness and kurtosis. Subsequently, the factorial structure of the scales was tested with CFA. The measurement model contained 17 factors (i.e., participation, feedback, autonomy, mental demands, time pressure, emotional demands, social support, competition, mobbing, physical burden, risk of accident, training opportunities, career advancement, job security, employability, work life conflict, and income satisfaction) that were allowed to correlate. We tested the factorial structure for each subgroup separately to see if the factor model adequately fitted across all subgroups in order to evaluate more stringent measurement invariance models in the next steps [107]. The MLR χ^2^-test statistic with robust standard errors [108] was calculated because it provides more accurate parameter estimations for items with five answer categories and for distortion from univariate and multivariate normality [109]. We fixed the factor variance to 1 and the factor mean to 0 for scale setting. We calculated the root mean squared error of approximation (*RMSEA*), standardized root mean square residual (*SRMR*), comparative fit index (*CFI*), and Tucker–Lewis index (*TLI*) to gauge model fit. For the *RMSEA* values between 0.05 and 0.08 indicate acceptable and values between 0.02 and 0.05 indicate good model fit. For the *CFI* and *TLI* values between 0.90 and 0.95 indicate acceptable and values between 0.95 and 0.99 indicate good model fit [110]. We used multigroup CFA (MGCFA) [110] to test for MI between the different language versions. Again, the fixed-factor method was used for scale setting as it has been found to be the best method to identify non-invariant indicators [111]. For the first group the factor mean is fixed to 0 and the factor variance is fixed to 1 while both are freed in all other groups in the metric and scalar invariance models. The Δ*CFI* was used to assess goodness of fit of MI models as it has been found to perform reasonably well in detecting (lack of) measurement invariance [112,113,114]. A Δ*CFI* > −0.01 between a baseline model and the resulting model indicates measurement invariance [110]. Full information maximum likelihood was used to account for missing values in CFA [115]. Criterion validity was assessed with intercorrelations (Pearson’s *r*). Furthermore, hierarchical regression analyses were used to investigate the predictive power of the QoW and QoE scales on the different forms of employees’ well-being. We included the variables in a stepwise manner in order to evaluate the incremental validity of the new scales. The first model included only demographic variables as control variables. In a next step, working time conditions were included as a second block of control variables. The third step included the QoE and the fourth step the QoW scales. We opted for this order because the first job quality indices mainly contained quality of employment measures [5]. Thus, we wanted to investigate the incremental contribution of the quality of work measures. R version 4.0.2 [116] was used for data analyses. Particularly, the mice package [117] was used for multiple imputation and the miceadds [118] and the naniar [119] packages for additional missing value analyses. The lavaan [120] and semTools [121] packages were used for the CFA and measurement invariance analyses. Finally, graphs were created with the ggplot2 package [122].

## 3. Results

### 3.1. Preliminary Analysis

Table 3 shows coefficients that describe the univariate distribution of each item. Item means ranged from 1.24 to 4.05 (*SD* between 0.59 and 1.41), skewness between −0.82 and 3.00, and kurtosis between −1.19 and 10.46. The percentage of missing values ranged from 0.07 to 5.32. Furthermore, items violated multivariate normality (Mardia’s multivariate skewness: ${\hat{\gamma }}_{1,43}$ = 116.4; χ^2^ = 29,526.40; *p* < 0.001; Mardia’s multivariate kurtosis: ${\hat{\gamma }}_{2,43}$ = 2224.78; *z* = 90.86; *p* < 0.001).

### 3.2. Factor Structure

**Figure 1:**
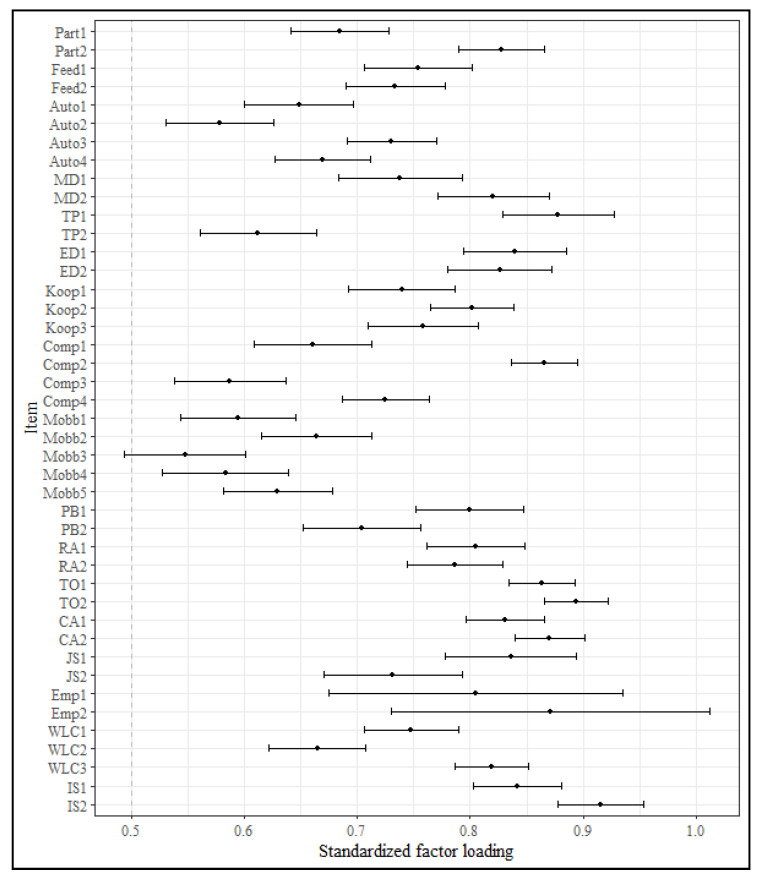
Standardized factor loadings with 95% confidence intervals for the total sample. Each item is specified to only load on its respective factor.

Table 4 shows the fit indices for the proposed factor models for the total sample and the different language versions. The fit indices for the total sample as well as the Luxembourgish and French version showed acceptable to good model fit. Only the *TLI* of the German version is somewhat below the cutoff and might be classified as mediocre [110]. Moreover, Table 4 shows the fit indices for the different measurement invariance models. According to Δ*CFI*, metric, as well as scalar, invariance between the language versions is confirmed. Figure 1 shows the standardized factor loadings for the total sample.

### 3.3. Internal Consistencies

Table 5 shows the internal consistencies of the QoW and QoE scales as well as the well-being scales. In the whole sample, the Cronbach’s α of the QoW and QoE scales ranged between 0.70 and 0.86. For the Luxembourgish version of these scales Cronbach’s α ranged between 0.63 and 0.87, for the French version between 0.67 and 0.88 and for the German version between 0.72 and 0.90. Thus, (with some exceptions) the reliability for the QoW and QoE scales can be deemed satisfactory. Regarding the outcome scales, only vigor (Cronbach’s α between 0.64 and 0.72) and performance (Cronbach’s α between 0.51 and 0.66) showed somewhat low reliabilities.

### 3.4. Intercorrelations

Table 6 shows the intercorrelations between the different QoW and QoE scales, the composite QoE and QoW and the different outcome measures. By trend, scales within an area were stronger correlated than scales between areas. For instance, participation and feedback, as well as mental demands and time pressure, or training opportunities and career advancement were moderately to strongly correlated. Mobbing showed strong positive correlations with burnout and health problems and negative correlations with work satisfaction, general well-being and vigor. The QoW is correlated with all well-being and health measures (*r* between |0.38| and |0.60|), as well as with work satisfaction (*r* = 0.60), vigor (*r* = 0.32), and subjective work performance (*r* = 0.11). The QoE is also correlated with all well-being and health measures (*r* between |0.31| and |0.44|), as well as with work satisfaction (*r* = 0.53), vigor (*r* = 0.31), and subjective work performance (*r* = 0.10).

### 3.5. Multiple Regression Analyses

Table 7, Table 8 and Table 9 show the results of the hierarchical regression analysis with z-standardized predictor and outcome variables. The first step only included the control variables (i.e., gender, age, nationality, residence, education, work sector). In a second step, long work week and atypical working time was included. The third step included the QoE scales. Finally, including the QoW scales in a fourth step increased *R*^2^ for all outcome variables significantly. The change in *R*^2^ ranged between 0.06 and 0.17. Cooperation was the strongest predictor for work satisfaction, while work life conflict was the strongest predictor for burnout, general well-being, and vigor. Health problems were best predicted by physical burden. Mobbing was consistently among the strongest predictors for all well-being scales.

## 4. Discussion

A review of job quality approaches has shown the need for a new questionnaire that covers the most recent conceptualization of job quality. Moreover, our aim was to connect the JD-R model that is well established in work psychology [42,43,44,45,46,47] with the job quality debate [5,6,7,8,9,10,11,12,13,14,15,16,17,18,19,20]. The proposed two job quality indices (i.e., QoW and QoE) contain job characteristics that are linked to well-being and/or health outcomes, and are rooted in the JD-R model. The new questionnaire showed good psychometric properties in a representative sample of employees working in Luxembourg. Thus, our sample covered a wide range of different occupations, making our results more generalizable. CFA indicated that the proposed factor model adequately fit to the data for the whole sample as well as for the Luxembourgish and French language versions. The German language version showed a slightly worse model fit. However, measurement invariance testing revealed scalar invariance between the language versions. Thus, the language versions can be used to compare employees across different language contexts [38]. Reliability analyses for the QoW and QoE scales showed generally acceptable Cronbach’s α (ranging from 0.70 to 0.87). The intercorrelations of the QoW scales ranged between |0.01| and |0.58| and the intercorrelations of the QoE between |0.02| and |0.57|. Bivariate analyses revealed that social support and mobbing were strongly associated with work satisfaction, while mobbing and work life conflict were strongly associated with burnout. Additionally, it was shown that mobbing and work life conflict were strong predictors for all well-being outcome variables. This is in line with recent meta-analyses that linked mobbing exposure to several negative outcomes, such as work satisfaction, burnout, general well-being, work motivation and physical health problems [64,123]. Some studies found that workplace mobbing exposure was the strongest workplace stressor [124] with devastating effects for exposed employees’ well-being and health [64,125]. Notably, mobbing may also affect other work characteristics, as it tends to decrease job and personal resources [126], inhibits cooperation, and decreases job satisfaction [127]. On the other hand, work life conflict (or low levels of work life balance and related constructs) have also been linked to several negative consequences [77,128]. Overall, the measures we introduced seem to account for a substantial amount of variance (*R*^2^ between 0.20 and 0.53) in the different aspects of well-being of employees in Luxembourg, as defined by work satisfaction, burnout, general well-being, vigor, and subjective physiological health problems and, thus, represents an important addition to the prevailing job quality research. Especially the work-specific well-being dimensions (i.e., burnout, job satisfaction) were well predicted by the QoW and QoE scales (*R*^2^ = 0.52, *R*^2^ = 0.53), corroborating the construct validity of these scales. Overall, the findings confirm the feasibility to assess the multi-dimensional conceptualization of job quality, based on a theoretical approach (JD-R- model) as well as on a pragmatic approach (EWCS-Survey). Especially the scalar invariance between the language versions makes the QoW and QoE scales particularly useful for international comparisons, and the verification of cultural differences.

The analyses and results presented in this article have some limitations. First of all, one of the developed scales, namely time pressure, was not as reliable as desired. However, all in all the internal consistencies of the QoW and QoE scales were above the often expressed cutoff value of 0.70 [129,130]. It is also important to note that the performance scale had a quite low internal consistency, especially for the German language version. Thus, the results of the correlation and regression analysis should be regarded with cautious regarding this outcome variable as performance showed a high standard error of measurement. Second, because of the cross-sectional design of the study, all correlations between the QoW scales and the different well-being measures cannot be interpreted in a causal manner. It is possible that employees with low levels of well-being perceived their working conditions worse than employees with high levels of well-being [131]. Third, it is important to note, that the mode of data collection can have an impact on factor structure, internal consistencies and intercorrelations of multi-item measures [132]. Thus, it is important that future studies investigate the psychometric properties of these scales for other data collection modes (e.g., online surveys, paper-pencil) and test its measurement invariance [132,133]. Future research might also expand the investigation on the reliability and validity of the QoW and QoE scales (e.g., test-retest-reliability, expansion of the nomological net). Moreover, longitudinal data with information about quality of work as well as quality of employment and health outcomes across time would be helpful to further validate the two composite indices in this respect. Future studies might also apply the new questionnaire within other countries to enable cross-national research.

## 5. Conclusions

A literature review revealed that despite the current availability of several indices of job quality, there is still a need of a worker-oriented, individually constructed and theoretically grounded job quality index. The new developed questionnaire shows great potential in measuring working conditions linked to well-being. So far it has been shown that it can identify important predictors of employees’ well-being and that it can be used to map the current job quality of employees. Two meaningful aggregate indices are now accessible, which are based on a system of well-defined measures of different attributes of work that have an impact on the well-being of employees.

## Figures and Tables

**Table 1:** Areas and dimensions of the Quality of Work Index Luxembourg in comparison with job quality (EWCS; Eurofound, 2016).

Job Quality (EWCS; Eurofound, 2016)		QoW and QoE	
Area	Dimension	Area	Dimension
Skills and discretion	Cognitive dimension Decision latitude Organizational participation Training	Job design (resources)	Participation Feedback Autonomy
Work intensity	Quantitative demands Pace determinants and interdependency Emotional demands	Work intensity (demands)	Mental demands Time pressure Emotional demands
Social environment	Adverse social behavior Social support Management quality	Social conditions	Social support Competition Mobbing
Physical environment	Posture related Ambient Biological & chemical	Physical conditions	Physical burden Risk of accident
Working time quality (objective)	Duration Atypical working time Working time arrangements Flexibility	Working time index	Items
Prospects	Employment status Career prospects Job security Downsizing	Quality of employment	Training opportunities Career advancement Job security Employability
Earnings (objective)	wages		Income satisfaction

**Table 2:** Sample characteristics.

	*n* (%)	Total	Language Version			Differences
			Luxembourgish	French	German	
Gender	Men	871 (57.2)	340 (51.4)	399 (61.5)	106 (61.6)	χ^2^_(2)_ = 15.406 **
	Women	651 (42.8)	322 (48.6)	250 (38.5)	66 (38.4)	
Age *M* (*SD*)		46.2 (9.0)	45.8 (9.7)	46.3 (8.4)	47.5 (8.4)	*F*_(1, 1481)_ = 4.801 *
Nationality	Luxembourgish	606 (39.9)	580 (87.7)	20 (3.1)	6 (3.5)	χ^2^_(8)_ = 2332.5 ***
	French	372 (24.5)	18 (2.7)	352 (54.3)	2 (1.2)	
	German	156 (10.3)	4 (0.6)	1 (0.2)	151 (87.8)	
	Belgian	185 (12.2)	12 (1.8)	166 (25.6)	7 (4.1)	
	Other	201 (13.2)	47 (7.1)	109 (16.8)	6 (3.5)	
Residence	Luxembourg	913 (60)	643 (97.1)	209 (32.2)	24 (14)	χ^2^_(6)_ = 1781.6 ***
	France	301 (19.8)	12 (1.8)	289 (44.5)	0 (0)	
	Germany	149 (9.8)	0 (0)	7 (1.1)	140 (81.4)	
	Belgium	159 (10.4)	7 (1.1)	144 (22.2)	8 (4.7)	
Education	ISCED 1	46 (3)	13 (2)	29 (4.5)	2 (1.2)	χ^2^_(14)_ = 49.895 ***
	ISCED 2	142 (9.4)	68 (10.3)	50 (7.7)	11 (6.4)	
	ISCED 3	549 (36.2)	279 (42.4)	201 (31)	65 (37.8)	
	ISCED 4	106 (7)	45 (6.8)	43 (6.6)	16 (9.3)	
	ISCED 5	108 (7.1)	34 (5.2)	57 (8.8)	15 (8.7)	
	ISCED 6	236 (15.5)	105 (16)	110 (16.9)	16 (9.3)	
	ISCED 7	303 (20)	101 (15.3)	148 (22.8)	43 (25)	
	ISCED 8	28 (1.8)	13 (2)	11 (1.7)	4 (2.3)	
Work sector (Isco-08)	Armed forces	2 (0.1)	2 (0.3)	0 (0)	0 (0)	χ^2^_(18)_ = 62.008 ***
	Managers	75 (5)	30 (4.6)	36 (5.6)	6 (3.5)	
	Professional	426 (28.3)	184 (28.2)	179 (27.8)	52 (30.2)	
	Technicians	377 (25)	189 (28.9)	134 (20.8)	48 (27.9)	
	Clerical support worker	186 (12.4)	95 (14.5)	70 (10.9)	21 (12.2)	
	Service and sales	146 (9.7)	62 (9.5)	66 (10.3)	15 (8.7)	
	Agricultural	14 (0.9)	10 (1.5)	3 (0.5)	1 (0.6)	
	Craft and related trades workers	151 (10)	47 (7.2)	73 (11.4)	25 (14.5)	
	Plant and machine operators	64 (4.3)	16 (2.5)	42 (6.5)	2 (1.2)	
	Elementary occupations	64 (4.3)	18 (2.8)	40 (6.2)	2 (1.2)	

* *p* < 0.05, ** *p* < 0.01, *** *p* < 0.001.

**Table 3:** Item characteristics.

Item	% Missing	Mean	SD	Skewness	Kurtosis
Part1	0.39	2.82	1.13	0.00	−0.74
Part2	0.85	3.15	1.04	−0.33	−0.32
Feed1	1.38	3.29	1.04	−0.31	−0.37
Feed2	1.64	2.99	1.11	−0.13	−0.73
Auto1	0.39	3.61	1.05	−0.57	−0.11
Auto2	0.07	2.70	1.30	0.13	−1.10
Auto3	0.07	3.52	1.16	−0.55	−0.41
Auto4	0.59	2.85	1.16	0.07	−0.75
MD1	0.00	4.05	0.79	−0.82	1.25
MD2	0.07	3.85	0.84	−0.66	0.62
TP1	0.13	3.21	1.11	−0.19	−0.64
TP2	0.26	3.65	1.06	−0.59	−0.24
ED1	0.33	3.15	1.23	−0.13	−0.97
ED2	0.46	2.82	1.26	0.08	−1.02
Sup1	4.20	3.68	0.90	−0.56	0.31
Sup2	4.27	3.96	0.83	−0.81	1.12
Sup3	4.20	3.88	0.93	−0.82	0.66
Comp1	3.94	1.99	0.99	0.82	0.15
Comp2	5.32	2.25	1.06	0.52	−0.48
Comp3	4.34	2.43	1.20	0.46	−0.78
Comp4	4.60	2.22	1.05	0.58	−0.27
Mobb1	1.31	2.11	0.83	0.58	0.39
Mobb2	1.18	1.67	0.89	1.39	1.67
Mobb3	1.12	1.86	0.96	0.97	0.28
Mobb4	1.18	1.24	0.59	3.00	10.46
Mobb5	1.12	1.83	0.80	0.71	0.04
PB1	0.07	2.72	1.40	0.36	−1.16
PB2	0.13	2.76	1.15	0.18	−0.76
RA1	0.07	2.01	1.14	0.89	−0.19
RA2	0.07	2.10	1.09	0.71	−0.37
TO1	0.85	2.92	1.23	−0.10	−0.98
TO2	1.31	3.02	1.28	−0.21	−1.04
CA1	2.43	2.21	1.10	0.55	−0.58
CA2	3.48	2.48	1.13	0.20	−0.89
JS1	0.46	3.90	1.03	−0.85	0.33
JS2	0.20	3.97	1.09	−0.89	0.05
Emp1	1.31	2.77	1.41	0.15	−1.24
Emp2	1.45	3.02	1.38	−0.04	−1.19
WLC1	0.13	2.33	1.08	0.49	−0.49
WLC2	0.20	2.29	1.05	0.41	−0.58
WLC3	0.07	2.28	0.98	0.45	−0.35
IS1	1.51	3.37	0.86	−0.27	0.27
IS2	0.26	3.38	0.85	−0.38	0.53

**Table 4:** Fit indices for single CFAs and measurement invariance across language version.

Version	χ^2^	df	RMSEA (90% CI)	CFI	TLI	SRMR
Total (*n* = 1522)	1896.339 ***	724	0.033 (0.031; 0.034)	0.943	0.929	0.038
Luxembourg (*n* = 662)	1377.066 ***	724	0.037 (0.034; 0.040)	0.924	0.905	0.043
French (*n* = 649)	1245.145 ***	724	0.033 (0.030; 0.036)	0.943	0.929	0.044
German (*n* = 172)	1012.422 ***	724	0.048 (0.041; 0.055)	0.915	0.893	0.058
Configural invariance	3697.663 ***	2172	0.038 (0.036; 0.040)	0.930	0.913	0.045
Metric invariance	3784.172 ***	2224	0.038 (0.036; 0.040)	0.929	0.913	0.048
Scalar invariance	4014.246 ***	2276	0.039 (0.037; 0.041)	0.921	0.906	0.049

RMSEA (90 CI) = root mean squared error of approximation with 90% confidence interval; CFI = comparative fit index; TLI = Tucker-Lewis index; SRMR = standardized root mean square residual; *** *p* < 0.001.

**Table 5:** Internal consistencies across the language versions.

Dimensions	Total	Luxembourgish	French	German
*Job design*				
Participation	0.72	0.75	0.67	0.78
Feedback	0.71	0.68	0.71	0.80
Autonomy	0.74	0.74	0.73	0.79
*Work intensity*				
Mental demands	0.75	0.74	0.77	0.76
Time pressure	0.70	0.63	0.74	0.74
Emotional demands	0.82	0.81	0.83	0.84
*Social conditions*				
Social support	0.80	0.79	0.79	0.81
Competition	0.78	0.77	0.77	0.83
Mobbing	0.73	0.74	0.70	0.82
*Physical conditions*				
Physical burden	0.71	0.69	0.73	0.72
Risk of accident	0.78	0.75	0.80	0.78
Quality of Employment				
Training opportunities	0.86	0.83	0.88	0.90
Career advancement	0.82	0.81	0.85	0.76
Job security	0.76	0.73	0.73	0.86
Employability	0.82	0.80	0.84	0.86
Work life conflict	0.78	0.75	0.80	0.81
Income satisfaction	0.86	0.87	0.85	0.89
*Outcomes*				
Burnout	0.83	0.83	0.83	0.87
General well-being	0.83	0.81	0.86	0.85
Subjective physiological health problems	0.72	0.71	0.73	0.75
Work satisfaction	0.83	0.83	0.82	0.81
Vigor	0.65	0.64	0.66	0.72
Performance	0.63	0.60	0.66	0.51

Cronbach’s α coefficients.

**Table 6:** Intercorrelations between study variables.

	**Variables**	***M***	***SD***	**1**	**2**	**3**	**4**	**5**	**6**	**7**	**8**	**9**	**10**	**11**	**12**
1	Long working hours ^a^	14%													
2	Atypical working hours ^a^	22%		0.14 ***											
3	Training opportunities	3.0	1.2	0.04	−0.04										
4	Career advancement	2.3	1.0	0.04	−0.02	0.58 ***									
5	Job security	3.9	1.0	−0.02	0.02	0.25 ***	0.22 ***								
6	Employability	2.9	1.3	0.01	0.02	0.16 ***	0.17 ***	0.19 ***							
7	Work life conflict	2.3	0.9	0.17 ***	0.18 ***	−0.03	−0.05 *	−0.20 ***	−0.02						
8	Income satisfaction	3.4	0.8	−0.01	0.00	0.29 ***	0.23 ***	0.31 ***	0.08 **	−0.11 ***					
9	QoE	3.2	0.6	−0.02	−0.05 *	0.70 ***	0.67 ***	0.60 ***	0.54 ***	−0.36 ***	0.53 ***				
10	Participation	3.0	1.0	0.10 ***	−0.06 *	0.27 ***	0.30 ***	0.21 ***	0.16 ***	−0.11 ***	0.23 ***	0.37 ***			
11	Feedback	3.1	0.9	−0.02	−0.06 *	0.28 ***	0.32 ***	0.12 ***	0.08 **	−0.11 ***	0.20 ***	0.32 ***	0.42 ***		
12	Autonomy	3.2	0.9	0.09 ***	−0.19 ***	0.18 ***	0.17 ***	0.16 ***	0.13 ***	−0.08 **	0.17 ***	0.26 ***	0.50 ***	0.22 ***	
13	Mental demands	4.0	0.7	0.14 ***	0.07 **	0.14 ***	0.06 *	0.02	0.07 **	0.25 ***	0.02	0.03	0.08 **	0.14 ***	0.07 **
14	Time pressure	3.4	1.0	0.20 ***	0.11 ***	−0.06 *	−0.03	−0.15 ***	0.00	0.36 ***	−0.17 ***	−0.20 ***	−0.08 ***	−0.01	−0.11 ***
15	Emotional demands	3.0	1.1	0.08 **	0.17 ***	0.04	−0.06 *	−0.12 ***	−0.04	0.32 ***	−0.03	−0.14 ***	−0.09 ***	−0.07 **	−0.12 ***
16	Social support	3.8	0.8	−0.01	0.00	0.23 ***	0.20 ***	0.18 ***	0.10 ***	−0.16 ***	0.24 ***	0.31 ***	0.27 ***	0.45 ***	0.17 ***
17	Competition	2.2	0.9	0.07 **	0.03	−0.02	0.03	−0.22 ***	−0.04	0.28 ***	−0.12 ***	−0.17 ***	−0.07 **	−0.10 ***	−0.06 *
18	Mobbing	1.7	0.6	0.08 **	0.05 *	−0.12 ***	−0.17 ***	−0.22 ***	−0.11 ***	0.35 ***	−0.18 ***	−0.31 ***	−0.33 ***	−0.32 ***	−0.21 ***
19	Physical burden	2.7	1.1	0.02	0.26 ***	−0.15 ***	−0.14 ***	−0.17 ***	−0.05 *	0.13 ***	−0.21 ***	−0.24 ***	−0.19 ***	−0.14 ***	−0.32 ***
20	Risk of accident	2.1	1.0	0.04	0.29 ***	−0.09 ***	−0.05 *	−0.06 *	−0.01	0.09 ***	−0.17 ***	−0.12 ***	−0.13 ***	−0.09 ***	−0.27 ***
21	QoW	3.3	0.4	−0.08 **	−0.26 ***	0.23 ***	0.25 ***	0.29 ***	0.12 ***	−0.40 ***	0.31 ***	0.44 ***	0.56 ***	0.48 ***	0.55 ***
22	Burnout	2.4	0.8	0.08 **	0.12 ***	−0.15 ***	−0.21 ***	−0.30 ***	−0.12 ***	0.50 ***	−0.31 ***	−0.43 ***	−0.29 ***	−0.23 ***	−0.23 ***
23	General well-being	3.2	1.0	−0.02	−0.02	0.13 ***	0.15 ***	0.23 ***	0.09 ***	−0.35 ***	0.17 ***	0.31 ***	0.24 ***	0.20 ***	0.18 ***
24	Health problems	2.1	0.7	0.00	0.05 *	−0.13 ***	−0.16 ***	−0.22 ***	−0.19 ***	0.31 ***	−0.22 ***	−0.35 ***	−0.23 ***	−0.18 ***	−0.17 ***
25	Work satisfaction	3.6	0.8	−0.04	−0.08 **	0.32 ***	0.31 ***	0.37 ***	0.15 ***	−0.36 ***	0.40 ***	0.53 ***	0.43 ***	0.38 ***	0.31 ***
26	Vigor	3.4	0.7	−0.01	0.00	0.13 ***	0.15 ***	0.22 ***	0.13 ***	−0.28 ***	0.19 ***	0.31 ***	0.26 ***	0.22 ***	0.18 ***
27	Performance	3.7	0.7	0.02	0.00	0.03	0.07 **	0.10 ***	0.08 **	−0.06 *	0.01	0.10 ***	0.19 ***	0.15 ***	0.15 ***
		**13**	**14**	**15**	**16**	**17**	**18**	**19**	**20**	**21**	**22**	**23**	**24**	**25**	**26**
14	Time pressure	0.38 ***													
15	Emotional demands	0.26 ***	0.31 ***												
16	Social support	0.11 ***	−0.11 ***	−0.05 *											
17	Competition	0.07 **	0.23 ***	0.21 ***	−0.21 ***										
18	Mobbing	0.07 **	0.21 ***	0.26 ***	−0.27 ***	0.34 ***									
19	Physical burden	−0.06 *	0.15 ***	0.15 ***	−0.10 ***	0.09 ***	0.20 ***								
20	Risk of accident	0.01	0.11 ***	0.12 ***	−0.03	0.10 ***	0.16 ***	0.58 ***							
21	QoW	−0.22 ***	−0.50 ***	−0.52 ***	0.44 ***	−0.43 ***	−0.57 ***	−0.58 ***	−0.52 ***						
22	Burnout	0.22 ***	0.35 ***	0.39 ***	−0.30 ***	0.28 ***	0.45 ***	0.37 ***	0.23 ***	−0.61 ***					
23	General well-being	−0.06 *	−0.22 ***	−0.23 ***	0.29 ***	−0.17 ***	−0.32 ***	−0.15 ***	−0.07 **	0.38 ***	−0.53 ***				
24	Health problems	0.06 *	0.19 ***	0.23 ***	−0.22 ***	0.17 ***	0.30 ***	0.33 ***	0.18 ***	−0.42 ***	0.55 ***	−0.43 ***			
25	Work satisfaction	−0.02	−0.28 ***	−0.26 ***	0.44 ***	−0.28 ***	−0.48 ***	−0.27 ***	−0.19 ***	0.60 ***	−0.58 ***	0.44 ***	−0.41 ***		
26	Vigor	−0.03	−0.14 ***	−0.16 ***	0.24 ***	−0.13 ***	−0.30 ***	−0.08 **	−0.05 *	0.32 ***	−0.45 ***	0.51 ***	−0.32 ***	0.44 ***	
27	Performance	0.08 **	0.08 **	−0.01	0.03	−0.02	−0.15 ***	−0.05 *	−0.05 *	0.11 ***	−0.12 ***	0.14 ***	−0.06 *	0.12 ***	0.20 ***

* *p* < 0.05, ** *p* < 0.01, *** *p* < 0.001. ^a^ higher values depict long working hours and atypical working hours.

**Table 7:** Hierarchical regression analyses with burnout and general well-being as outcome variables.

Predictor Variables	Burnout				General Well-Being			
	Step 1	Step 2	Step 3	Step 4	Step 1	Step 2	Step 3	Step 4
Demographic variables								
Long work week		0.19 *	0.00	−0.04		−0.08	0.05	0.06
Atypical working time		0.26 ***	0.09	−0.10 *		−0.04	0.08	0.13 *
Training opportunities			0.03	0.01			0.03	0.01
Career advancement			−0.11 ***	−0.05 *			0.06 *	0.02
Job security			−0.13 ***	−0.06 **			0.12 ***	0.08 **
Employability			−0.05 *	−0.04 *			0.04	0.03
Work life conflict			0.45 ***	0.25 ***			−0.34 ***	−0.22 ***
Income satisfaction			−0.20 ***	−0.12 ***			0.07 **	0.02
Participation				−0.07 **				0.06 *
Feedback				0.01				0.01
Autonomy				0.00				0.02
Mental demands				0.12 ***				0.00
Time pressure				0.05 *				−0.06 *
Emotional demands				0.16 ***				−0.09 ***
Social support				−0.13 ***				0.16 ***
Competition				0.02				0.02
Mobbing				0.15 ***				−0.11 ***
Physical burden				0.23 ***				−0.05
Risk of accident				0.01				0.04
*F*	2.085	4.918 ***	57.223 ***	63.291 ***	1.016	0.987	22.472 ***	19.295 ***
Δ*F*		12.928 ***	124.417 ***	46.687 ***		0.902	50.790 ***	12.805 ***
*R* ^2^	0.01	0.03	0.35	0.52	0.00	0.01	0.17	0.24
Δ*R*^2^		0.02	0.32	0.17		0.00	0.16	0.07

* *p* < 0.05, ** *p* < 0.01, *** *p* < 0.001; standardized regression coefficients, the block demographic variables contains gender, age, nationality, residence, education, work sector; *F* represents the test against the null model (all coefficients equal zero), Δ*F* represents the test against the regression model of the previous step.

**Table 8:** Hierarchical regression analyses with subjective physiological health problems and work satisfaction as outcome variables.

Predictor Variables	Subjective Physiological Health Problems				Work Satisfaction			
	Step 1	Step 2	Step 3	Step 4	Step 1	Step 2	Step 3	Step 4
Demographic variables								
Long work week		0.10	−0.04	−0.07		−0.07	0.06	0.04
Atypical working time		0.11	−0.01	−0.14 *		−0.19 **	−0.06	0.03
Training opportunities			0.02	0.02			0.09 ***	0.06 **
Career advancement			−0.04	0.00			0.12 ***	0.05 *
Job security			−0.11 ***	−0.06 *			0.17 ***	0.11 ***
Employability			−0.09 ***	−0.09 ***			0.03	0.01
Work life conflict			0.32 ***	0.20 ***			−0.31 ***	−0.14 ***
Income satisfaction			−0.13 ***	−0.08 **			0.25 ***	0.17 ***
Participation				−0.04				0.14 ***
Feedback				−0.02				0.06 *
Autonomy				0.04				0.05 *
Mental demands				0.04				0.01
Time pressure				0.03				−0.07 **
Emotional demands				0.08 **				−0.09 ***
Social support				−0.08 **				0.18 ***
Competition				0.02				−0.04 *
Mobbing				0.09 ***				−0.17 ***
Physical burden				0.23 ***				−0.04
Risk of accident				0.00				−0.03
*F*	12.662 ***	10.151 ***	30.844 ***	25.817 ***	6.601 ***	6.306 ***	61.073 ***	66.431 ***
Δ*F*		2.732	55.662 ***	15.363 ***		5.348 **	130.299 ***	46.934 ***
*R* ^2^	0.05	0.05	0.22	0.30	0.03	0.03	0.36	0.53
Δ*R*^2^		0.00	0.17	0.08		0.01	0.33	0.17

* *p* < 0.05, ** *p* < 0.01, *** *p* < 0.001; standardized regression coefficients, the block demographic variables contains gender, age, nationality, residence, education, work sector; *F* represents the test against the null model (all coefficients equal zero), Δ*F* represents the test against the regression model of the previous step.

**Table 9:** Hierarchical regression analyses with vigor and subjective work performance as outcome variables.

Predictor Variables	Vigor				Subjective Work Performance			
	Step 1	Step 2	Step 3	Step 4	Step 1	Step 2	Step 3	Step 4
Demographic variables								
Long work week		0.01	0.12	0.08		0.05	0.07	−0.02
Atypical working time		0.00	0.10	0.13 *		0.00	0.01	0.06
Training opportunities			0.02	0.00			−0.03	−0.04
Career advancement			0.06 *	0.01			0.08 *	0.02
Job security			0.12 ***	0.09 ***			0.08 **	0.07 *
Employability			0.08 **	0.06 *			0.09 **	0.06 *
Work life conflict			−0.27 ***	−0.19 ***			−0.06 *	−0.06
Income satisfaction				0.06 *			−0.04	−0.05
Participation				0.10 ***				0.12 ***
Feedback				0.04				0.08 *
Autonomy				0.06 *				0.08 *
Mental demands				0.01				0.05
Time pressure				−0.01				0.11 ***
Emotional demands				−0.06 *				0.01
Social support				0.10 ***				−0.06
Competition				0.03				0.01
Mobbing				−0.11 ***				−0.08 **
Physical burden				0.04				0.01
Risk of accident				0.00				−0.03
*F*	0.629	0.471	17.812 ***	14.995 ***	2.035	1.566	3.649 ***	5.735 ***
Δ*F*		0.005	40.823 ***	9.949 ***		0.183	6.394 ***	8.134 ***
*R* ^2^	0.00	0.00	0.14	0.20	0.01	0.01	0.03	0.09
Δ*R*^2^		0.00	0.14	0.06		0.00	0.02	0.06

* *p* < 0.05, ** *p* < 0.01, *** *p* < 0.001; standardized regression coefficients, the block demographic variables contains gender, age, nationality, residence, education, work sector; *F* represents the test against the null model (all coefficients equal zero), Δ*F* represents the test against the regression model of the previous step.

## Appendix A

**Table A1:** Quality of Work items.

Area	Dimension	Variable	Item	Answer Category
Job design	Participation	Part1	To what extent are you involved in decisions in your organisation?	1 (= *to a very low extent*) to 5 (= *to a very large extent*)
		Part2	To what extent does your superior consider your opinion in decisions or in upcoming changes?	1 (= *to a very low extent*) to 5 (= *to a very large extent*)
	Feedback	Feed1	To what extent do you receive feedback about your work from your superior or from your colleagues?	1 (= *to a very low extent*) to 5 (= *to a very large extent*)
		Feed2	To what extent do you receive feedback from your superior about your professional competences?	1 (= *to a very low extent*) to 5 (= *to a very large extent*)
	Autonomy	Auto1	To what extent can you decide how you carry out your work?	1 (= *to a very low extent*) to 5 (= *to a very large extent*)
		Auto2	To what extent can you determine your working hours yourself?	1 (= *to a very low extent*) to 5 (= *to a very large extent*)
		Auto3	To what extent can you determine the order of your work tasks yourself?	1 (= *to a very low extent*) to 5 (= *to a very large extent*)
		Auto4	To what extent can you determine the content of your work yourself?	1 (= *to a very low extent*) to 5 (= *to a very large extent*)
Work intensity	Mental demands	MD1	To what extent does your work demand concentration?	1 (= *to a very low extent*) to 5 (= *to a very large extent*)
		MD2	To what extent is your work intellectually challenging?	1 (= *to a very low extent*) to 5 (= *to a very large extent*)
	Time pressure	TP1	How often are you under time pressure or rushed in your work?	1 (= *never*) to 5 (= (*almost*) *always*)
		TP2	How often are you required to meet tight deadlines in your work?	1 (= *never*) to 5 (= (*almost*) *always*)
	Emotional demands	ED1	How often does your work require you to control your feelings?	1 (= *never*) to 5 (= (*almost*) *always*)
		ED2	How often does your work require you to hide your true feelings?	1 (= *never*) to 5 (= (*almost*) *always*)
Social conditions	Social support	Sup1	To what extent are you supported in your work by your colleagues?	1 (= *to a very low extent*) to 5 (= *to a very large extent*)
		Sup2	To what extent do you and your colleagues help one another with work-related problems?	1 (= *to a very low extent*) to 5 (= *to a very large extent*)
		Sup3	To what extent are you able to ask your colleagues for help for work-related problems?	1 (= *to a very low extent*) to 5 (= *to a very large extent*)
	Competition	Comp1	To what extent are you competing with your colleagues?	1 (= *to a very low extent*) to 5 (= *to a very large extent*)
		Comp2	To what extent is there any competition amongst your colleagues?	1 (= *to a very low extent*) to 5 (= *to a very large extent*)
		Comp3	To what extent is there competitive pressure in your work area?	1 (= *to a very low extent*) to 5 (= *to a very large extent*)
		Comp4	To what extent are there rivalries in your group of colleagues?	1 (= *to a very low extent*) to 5 (= *to a very large extent*)
	Mobbing	Mobb1	How often is your work criticised by your colleagues or by your superior?	1 (= *never*) to 5 (= (*almost*) *always*)
		Mobb2	How often are you ignored at work by your colleagues or your superior?	1 (= *never*) to 5 (= (*almost*) *always*)
		Mobb3	How often are you assigned meaningless tasks by your superior?	1 (= *never*) to 5 (= (*almost*) *always*)
		Mobb4	How often are you ridiculed in front of others by your superior or by your colleagues?	1 (= *never*) to 5 (= (*almost*) *always*)
		Mobb5	How often are you in conflict with your colleagues or superior?	1 (= *never*) to 5 (= (*almost*) *always*)
Physical conditions	Physical burden	PB1	How often is your work physically strenuous, e.g., does it involve prolonged standing?	1 (= *never*) to 5 (= (*almost*) *always*)
		PB2	How often does your work leave you physically exhausted?	1 (= *never*) to 5 (= (*almost*) *always*)
	Risk of accident	RA1	To what extent does your work put you at risk of accident and injury?	1 (= *to a very low extent*) to 5 (= *to a very large extent*)
		RA2	To what extent is your work carried out in working conditions that are harmful to health?	1 (= *to a very low extent*) to 5 (= *to a very large extent*)

**Table A2:** Quality of Employment items.

Area	Variable	Item	Answer Category
Training opportunities	TO1	To what extent do you have possibilities to engage in further training in your organization?	1 (= *to a very low extent*) to 5 (= *to a very large extent*)
	TO2	To what extent does your organization support you to undertake further training?	1 (= *to a very low extent*) to 5 (= *to a very large extent*)
Career advancement	CA1	To what extent do you have possibilities of advancement and promotion in your organization?	1 (= *to a very low extent*) to 5 (= *to a very large extent*)
	CA2	To what extent does your organization support professional advancement or promotion?	1 (= *to a very low extent*) to 5 (= *to a very large extent*)
Job security	JS1	To what extent do you consider your own job as being safe?	1 (= *to a very low extent*) to 5 (= *to a very large extent*)
	JS2	To what extent are you afraid to lose your job? (reversed)	1 (= *to a very low extent*) to 5 (= *to a very large extent*)
Employability	Emp1	How difficult would it be for you to find a similar job, if you were to lose or resign from your job? (reversed)	1 (= *not difficult at all*) to 5 (= *very difficult*)
	Emp2	And how difficult would it be for you to actually find a job, if you were to lose or resign from your job? (reversed)	1 (= *not difficult at all*) to 5 (= *very difficult*)
Work life conflict	WLC1	How often are you unable to reconcile your work and your private life?	1 (= *never*) to 5 (= (*almost*) *always*)
	WLC2	How difficult is it for you to give the necessary attention to your work as well as your private life?	1 (= *not difficult at all*) to 5 (= *very difficult*)
	WLC3	How often are conflicts arising as a result of the demands of your work and those of your private life?	1 (= *never*) to 5 (= (*almost*) *always*)
Income satisfaction	IS1	To what extent does your salary reflect your work input?	1 (= *to a very low extent*) to 5 (= *to a very large extent*)
	IS2	How satisfied are you at present with your salary?	1 (= *to a very low extent satisfied*) to 5 (= *to a very large extent satisfied*)

**Table A3:** Working time and atypical working hours.

Area	Variable	Item	Answer Category
Working time	WT	With reference to the last 12 months, how many hours a week do you work on average? Please include any regular additional time or overtime.	Open ended
Atypical working hours	AWH	How many days per month do you work in the evening from 7 PM or at night from 10 PM or at the weekend?	Open ended

**Table A4:** Outcome items.

Area	Variable	Item	Answer Category
Work satisfaction	WS1	How satisfied are you at present with your work?	1 (= *to a very low extent satisfied*) to 5 (= *to a very large extent satisfied*)
	WS2	How satisfied are you at present with the work atmosphere at work?	1 (= *to a very low extent satisfied*) to 5 (= *to a very large extent satisfied*)
	WS3	How satisfied are you at present with the working conditions at work?	1 (= *to a very low extent satisfied*) to 5 (= *to a very large extent satisfied*)
Burnout	Burn1	How often do you feel exhausted at the end of a working day?	1 (= *never*) to 5 (= (*almost*) *always*)
	Burn2	How often do you feel exhausted in the morning at the thought of a new working day?	1 (= *never*) to 5 (= (*almost*) *always*)
	Burn3	How often do you feel that every working hour is exhausting for you?	1 (= *never*) to 5 (= (*almost*) *always*)
	Burn4	To what extent is your work emotionally exhausting?	1 (= *to a very low extent*) to 5 (= *to a very large extent*)
	Burn5	To what extent are you frustrated by your work?	1 (= *to a very low extent*) to 5 (= *to a very large extent*)
	Burn6	To what extent Do you feel burnt out by your work?	1 (= *to a very low extent*) to 5 (= *to a very large extent*)
General well-being (WHO-5)	WHO1	In the last two weeks I have been happy and in a good mood.	1 (= *at no time*) to 5 (= *all the time*)
	WHO2	In the last two weeks I have felt calm and relaxed.	1 (= *at no time*) to 5 (= *all the time*)
	WHO3	In the last two weeks I have felt energetic and active.	1 (= *at no time*) to 5 (= *all the time*)
	WHO4	In the last two weeks I have woken up refreshed and well rested.	1 (= *at no time*) to 5 (= *all the time*)
	WHO5	In the last two weeks my day-to-day life has been busy with things that interest me.	1 (= *at no time*) to 5 (= *all the time*)
Subjective physiological health problems	SPHP1	How often have you experienced health problems in the last 12 months?	1 (= *never*) to 5 (= (*almost*) *always*)
	SPHP2	How often in the last 12 months have you had heart problems?	1 (= *never*) to 5 (= (*almost*) *always*)
	SPHP3	How often in the last 12 months have you had headaches?	1 (= *never*) to 5 (= (*almost*) *always*)
	SPHP4	How often in the last 12 months have you had back problems?	1 (= *never*) to 5 (= (*almost*) *always*)
	SPHP5	How often in the last 12 months have you had joint problems?	1 (= *never*) to 5 (= (*almost*) *always*)
	SPHP6	How often in the last 12 months have you had stomach problems?	1 (= *never*) to 5 (= (*almost*) *always*)
	SPHP7	How often in the last 12 months have you had insomnia?	1 (= *never*) to 5 (= (*almost*) *always*)
Vigor	Vigor1	How often do you have the feeling that you are overflowing with energy at work?	1 (= *never*) to 5 (= (*almost*) *always*)
	Vigor2	How often do you feel fit and vigorous at work?	1 (= *never*) to 5 (= (*almost*) *always*)
	Vigor3	How often do you look forward to going to work as you get up in the morning?	1 (= *never*) to 5 (= (*almost*) *always*)
Work performance	WP1	How do you evaluate your overall work performance in comparison with that of your colleagues?	1 (= *below average*) to 5 (= *above average*)
	WP2	How does your superior evaluate your overall work performance?	1 (= *below average*) to 5 (= *above average*)
